# A randomised controlled trial of the effects of albendazole in pregnancy on maternal responses to mycobacterial antigens and infant responses to bacille Calmette-Guérin (BCG) immunisation [ISRCTN32849447]

**DOI:** 10.1186/1471-2334-5-115

**Published:** 2005-12-21

**Authors:** Alison M Elliott, Proscovia B Namujju, Patrice A Mawa, Maria A Quigley, Margaret Nampijja, Peter M Nkurunziza, John T Belisle, Moses Muwanga, James AG Whitworth, the "Mother and Baby" study team

**Affiliations:** 1 Uganda Virus Research Institute, P.O. Box 49, Entebbe, Uganda; 2 Entebbe Hospital, P.O. Box 29, Entebbe, Uganda; 3 National Perinatal Epidemiology Unit, Oxford University, Old Road Campus, Headington, Oxford OX3 7LF, UK; 4 Department of Microbiology, Colorado State University, Fort Collins, Colorado 80523, USA; 5 London School of Hygiene & Tropical Medicine, Keppel Street, London WC1E 7HT, UK

## Abstract

**Background:**

Maternal schistosomiasis and filariasis have been shown to influence infant responses to neonatal bacille Calmette-Guérin (BCG) immunisation but the effects of maternal hookworm, and of de-worming in pregnancy, are unknown.

**Methods:**

In Entebbe, Uganda, we conducted a randomised, double-blind, placebo-controlled trial of a single dose of 400 mg of albendazole in the second trimester of pregnancy. Neonates received BCG. Interferon-gamma (IFN-γ) and interleukin (IL)-5 responses to a mycobacterial antigen (crude culture filtrate proteins (CFP) of *Mycobacterium tuberculosis*) were measured in a whole blood assay. We analysed results for binary variables using χ^2 ^tests and logistic regression. We analysed continuous variables using Wilcoxon's tests.

**Results:**

Maternal hookworm was associated with reduced maternal IFN-γ responses to CFP (adjusted odds ratio for IFN-γ > median response: 0.14 (95% confidence interval 0.02–0.83, p = 0.021). Conversely, maternal hookworm was associated with subsequent increased IFN-γ responses in their one-year-old infants (adjusted OR 17.65 (1.20–258.66; p = 0.013)). Maternal albendazole tended to reduce these effects.

**Conclusion:**

Untreated hookworm infection in pregnancy was associated with reduced maternal IFN-γ responses to mycobacterial antigens, but increased responses in their infants one year after BCG immunisation. The mechanisms of these effects, and their implications for protective immunity remain, to be determined.

## Background

Bacille Calmette-Guérin (BCG) immunisation is relatively ineffective against tuberculosis in the tropics, where the incidence of tuberculosis is high [1]. Helminth infection induces T-helper-2 responses and production of immunoregulatory cytokines and molecules that modulate responses to both helminths and bystander antigens; T-helper-1 and cytotoxic responses, required for immunity to viruses and intracellular bacteria, may be impaired [2]. Recognition of these effects has promoted speculation that helminth infection contributes to the observed reduced efficacy of BCG, and increased susceptibility to tuberculosis, in tropical countries [3]. BCG is often administered at birth in developing countries, so the observation that sensitisation to maternal schistosomiasis or filariasis in utero was associated with reduced interferon (IFN)-γ and increased interleukin (IL)-5 responses to mycobacterial antigens at one year of age, following BCG immunisation at birth, is of particular interest [4]. Hookworm is more widespread than schistosomiasis or filariasis [5], and may also suppress IFN-γ responses, both to hookworm, and to mycobacterial antigens [6]. Routine anthelminthic treatment after the first trimester of pregnancy, targeting hookworm anaemia, is now advocated, despite theoretical risks of teratogenicity (seen in animal models but not in man) [7], as benefits are expected to outweigh risks. However, the impact of this policy on other aspects of maternal and infant health has not been thoroughly investigated [8]. We wished to determine whether maternal hookworm infection had similar effects on neonatal responses to BCG to those reported for schistosomiasis and filariasis, and whether any such effects were removed by treatment for hookworm during pregnancy.

## Methods

Mothers in the second trimester of pregnancy were enrolled in a randomised, double-blind, placebo-controlled trial of albendazole treatment during pregnancy, at Entebbe Hospital, Uganda, between June and August 2002. The study was designed to examine effects of albendazole treatment in pregnancy on immunological and disease outcomes in infants. The design was revised when the World Health Organisation announced new recommendations for treatment of helminths in pregnancy [9]. This paper presents data for the preliminary group of mothers, enrolled before the change of protocol. Mothers were included if they were resident in the study area, planning to deliver in hospital and willing to know their HIV status. Mothers with haemoglobin below 8 g/dl were excluded and treated for hookworm and anaemia. Other exclusion criteria were abnormal pregnancy or history of adverse reaction to anthelminthic drugs. Before treatment, mothers were screened for intestinal parasites by the Kato-Katz method and stool culture [10]; blood was examined for malaria, microfilariae [11], full blood count and HIV (within a programme for prevention of mother-to-child HIV transmission). All eligible mothers were then randomised to treatment with single-dose albendazole (400 mg) or placebo, regardless of whether hookworm was detected or not. The randomisation sequence was generated by an independent statistician (MQ), using blocks of 50. Albendazole and matching placebo tablets (GlaxoSmithKline, UK) were packaged in identical envelopes, labelled with the randomisation code. Clinic staff gave participants the next number in the sequence, in order of enrolment.

At delivery mothers were asked to provide a second stool and blood sample; if possible, cord blood was obtained. Infants were immunised with intradermal BCG (100,000–330,000 colony forming units; Serum Institute of India, India). Six weeks after delivery all mothers were treated with both albendazole and praziquantel. Stool and blood samples were obtained from infants at one year of age.

All participants gave written informed consent. Ethical approval was given by ethics committees of the Uganda Virus Research Institute, Uganda National Council for Science and Technology and London School of Hygiene and Tropical Medicine.

### Immunological assays

The primary outcome measures were immune responses in mothers and their infants, measured as follows. We examined stimulated IFN-γ and IL-5 responses in a whole blood assay, and measured serum levels of regulatory cytokines (IL-10 and Transforming Growth Factor (TGF)-β). In the whole blood assay [12], unseparated, heparinised blood was diluted to a final concentration of one-in-four using RPMI supplemented with penicillin, streptomycin and glutamine, plated in 96-well plates, and stimulated with crude culture filtrate protein of *M. tuberculosis *(CFP) (5 μg/ml), or phytohaemagglutinin (PHA) (10 μg/ml) (Sigma, UK), or left unstimulated. Supernatants were harvested on day 6 and frozen until analysed.

Cytokine concentrations in serum or supernatants were measured by ELISA (Becton Dickinson, UK). The sensitivity of the assays was 62.5 pg/ml for TGF-β; 2.5 pg/ml for IL-10; 7.8 pg/ml for IL-5; 9.4 pg/ml for mothers and 37.5 pg/ml for infants for IFN-γ. Low-level cytokine production in un-stimulated wells was subtracted from concentrations produced in response to stimulation. Assays for samples with results above the highest standard were repeated after dilution.

### Statistical methods

Cytokine levels and responses were skewed so quantitative data were analysed using Wilcoxon's tests for paired or unpaired data as appropriate. A high proportion of mothers and infants showed positive IFN-γ responses to CFP. To allow analysis of effects of potential confounding factors using logistic regression, a binary variable was created indicating IFN-γ production below or above the median response. The sample size for this analysis was determined by the number of mothers recruited before the change in study protocol. Our analysis was not typical of a controlled trial, because our objective was to study both the effects of maternal helminths (by comparison of outcomes between mothers with and without helminths) and the modification of their effects by treatment (in the trial). Therefore, we first examined the effect of maternal helminth infection on cytokine responses in mothers at enrolment, and in their infants when they reached one year of age, and used the binary variable for high IFN-γ response to perform an analysis that adjusted for potential confounding factors using logistic regression. We then analysed the treatment trial by intention-to-treat: for each analysis, comparisons were made between albendazole and placebo recipients, first, for all participants, and second, for the sub-group of mothers with hookworm at enrolment (in whom any effect of treatment was expected to be greater). We examined the effect of treatment on responses in the mothers (using samples taken at delivery); in cord blood; and in one-year-old infants. Unblinded analyses were conducted by MQ. All other staff and participants remain blinded to treatment allocation as follow up continues.

## Results

**Figure 1:**
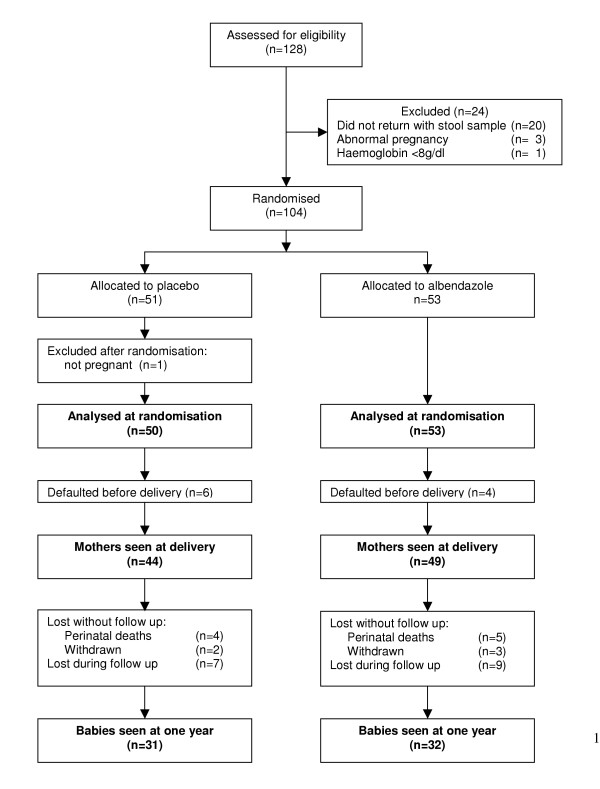
Study profile.

The trial profile is shown in figure 1. At enrolment, mothers' mean age was 23 years; 15/103 (15%) were HIV-positive; 15/103 (15%) had malaria; 38/101 (38%) had hookworm, 23/101 (23%) *Schistosoma mansoni*, 13/101 (13%) *Trichuris trichiura*, 6/101 (6%) *Ascaris lumbricoides*, 13/84 (15%) *Strongyloides stercoralis *and 21/99 (22%) *Mansonella perstans*. Of those with hookworm, 28 had light (1–999 eggs/g), eight moderate (1000–3999 eggs/g) and two heavy (≥ 4000 eggs/g) infection. Maternal helminth infection showed expected associations with younger age (p = 0.027) and lower education (p = 0.002). Mothers in albendazole and placebo groups were similar in age, HIV, malaria and helminth prevalence, and baseline cytokine levels and responses. After delivery the prevalence of hookworm was 0/47 (0%) in the albendazole group and 14/42 (33%) in the placebo group; other helminths remained similar in the two groups. Among one-year-olds, 3/63 had hookworm (one whose mother received albendazole, two placebo), 1/63 had *Trichuris *(albendazole-mother) and 1/54 *Mansonella *(placebo-mother).

### Immunological findings

Kato-Katz stool results for mothers at enrolment and all cytokine results were available for 90/103 mothers at enrolment, 79/93 at delivery (72 with results at both time points), and 57/63 one-year-olds. Whole blood assay data were missing when assays were unsatisfactory, and samples insufficient for repeats. Analysis of cord blood samples was restricted to 48/52 samples where mixing with maternal blood was excluded using an alkaline denaturation test [13]; serum cytokine results were complete for these 48 samples. Whole blood assay data were complete for only 33 of these cord blood samples: only five had a positive IFN-γ response, and one a positive IL-5 to CFP; therefore cord blood whole blood assay results are not presented further here.

### Effects of maternal helminth infection on cytokine responses in mothers at enrolment and in their one-year-old infants

**Figure 2:**
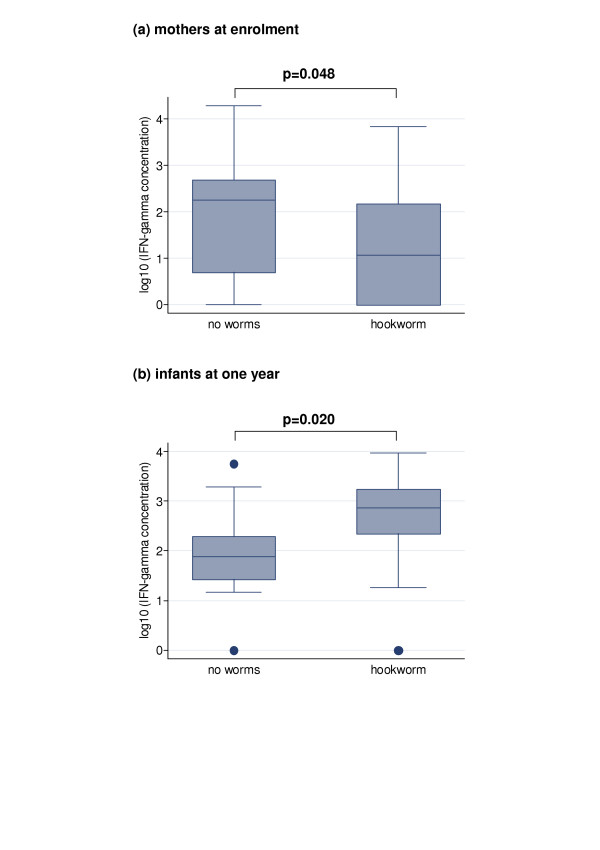
**Effects of maternal hookworm on responses mycobacterial antigens in mothers during pregnancy and their one-year-old infants**. Effects of maternal hookworm on IFN-γ responses to culture filtrate proteins of *Mycobacterium tuberculosis *in (a) mothers at enrolment during pregnancy (before receiving study treatment) and (b) their one-year-old infants, who received BCG immunisation at birth. IFN-γ responses shown are net production: low-level cytokine production in un-stimulated wells was subtracted from concentrations produced in response to stimulation to give a measure of antigen-specific response. "No worms": no helminths detected in stool or blood sample from mother either in pregnancy or at delivery. "Hookworm": mother had hookworm in pregnancy. Boxes show median value, 25th and 75th centiles; whiskers show lower and upper adjacent values and dots indicate outside values. P values <0.10 (Wilcoxon's rank sum test) are shown.

At enrolment, all helminth species were associated with lower maternal IFN-γ responses to CFP. This effect was most marked, and statistically significant, for hookworm (median 10 (interquartile range 0, 162) pg/ml compared to 173 (4, 471) pg/ml, p = 0.048) (Figure 2(a)). Five mothers had a positive IL-5 response to CFP; all had helminth infections.

Among one-year-old infants, the opposite effect was seen: IFN-γ responses to CFP were higher, for all species of helminth, among infants of mothers who had helminth infections in pregnancy. Despite effective treatment of maternal hookworm in approximately half the cases, this effect was also strongest for hookworm (median IFN-γ production 737 (220, 1805) pg/ml for infants of mothers with hookworm; 75 (24, 228) pg/ml for infants of mothers without helminths, p = 0.020) (Figure 2(b)). Nine infants had positive IL-5 responses to CFP; 6 had mothers with helminth infection.

It was considered possible that the associations between maternal hookworm and responses to CFP in the mothers, or in their infants, might be explained by confounding factors such as co-infection with other pathogens (maternal malaria or HIV), maternal age, socioeconomic factors, or maternal BCG immunisation. We investigated this by creating binary variables for IFN-γ production below and above the median level of response in mothers and infants. This allowed us to examine associations between such factors and responses to CFP (Table) and to adjust for their effects using logistic regression. The observed associations between maternal hookworm and IFN-γ responses to CFP in mothers and their infants were not explained by any of the variables examined: odds ratios for the associations between maternal hookworm and high IFN-γ responses to CFP, adjusted for maternal age, HIV status, malaria parasitaemia, education, BCG scar and schistosomiasis were, for mothers at enrolment: 0.14 (95% confidence interval 0.02–0.83, p = 0.021); for infants at one year of age: 17.65 (95% CI: 1.20–258.66, p = 0.013).

### Analysis of the trial: effects of maternal treatment with albendazole on cytokine responses to CFP

**Figure 3:**
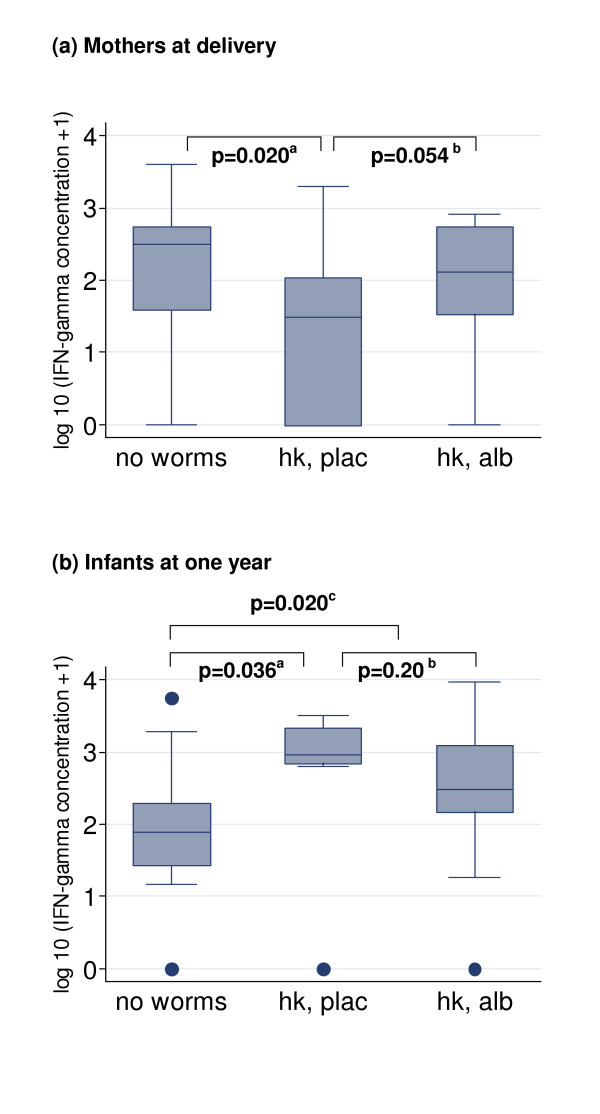
**Effects of maternal hookworm and albendazole treatment on responses mycobacterial antigens in mothers at delivery and their one-year-old infants**. Effects of maternal hookworm, and maternal treatment with albendazole during pregnancy, on IFN-γ responses to culture filtrate proteins of *Mycobacterium tuberculosis *in (a) mothers at delivery and (b) their one-year-old infants, who received BCG immunisation at birth. IFN-γ responses shown are net production: low-level cytokine production in un-stimulated wells was subtracted from concentrations produced in response to stimulation to give a measure of antigen-specific response. "No worms": no helminths detected in stool or blood sample from mother either in pregnancy or at delivery. "Hk, plac": mother had hookworm in pregnancy and received placebo. "Hk, alb": mother had hookworm in pregnancy and received albendazole. Boxes show median value, 25th and 75th centiles; whiskers show lower and upper adjacent values and dots indicate outside values. P values <0.10 (Wilcoxon's rank sum test) are shown comparing ^a ^no worms vs hookworm, placebo; ^b ^hookworm, placebo vs albendazole; ^c ^no worms vs hookworm, whether placebo or albendazole.

Mothers who received albendazole had an increase in IFN-γ response to CFP (median increase +26 (-7, +293) pg/ml), whereas placebo recipients did not (0 (-279, +73) pg/ml) (p = 0.019)). Among hookworm-infected mothers, corresponding increases were +55 (0, +386) pg/ml for albendazole, and 0 (-44, +81) pg/ml for placebo recipients (p = 0.055). Thus, treatment of hookworm in the mothers resulted in a profile of CFP responses that was similar to the "no helminth" group (Figure 3a). No effects of albendazole were observed on maternal IL-5 responses.

Conversely, IFN-γ responses to CFP were lower in infants of hookworm-infected mothers who received albendazole than in those whose mothers received placebo (Figure 3b) and IL-5 responses less frequent, although these effects were not statistically significant. The BCG scar diameter in one-year-old infants followed a similar pattern, being significantly smaller in infants of hookworm-infected mothers who received albendazole (2.0 (1.0, 3.0) mm) than in those whose mothers received placebo (3.5 (2.8, 4.0) mm; p = 0.026). BCG scar diameter showed a positive correlation with the IL-5 response to CFP (ρ = 0.32, p = 0.011) but not with the IFN-γ response.

### Non-specific immune responses

In most analyses, maternal helminths and albendazole treatment showed no effect on PHA responses or serum cytokine levels. However, cord blood IL-10 was lower for albendazole than placebo recipients, and this effect was statistically significant among hookworm mothers, (median 3 (0, 5) pg/ml versus 12 (4, 18) pg/ml, p = 0.031). TGF-β was detected in cord blood only for three mothers, all placebo recipients, of whom two were hookworm-infected.

## Discussion

We present results of a preliminary study, the principal limitations of which are small numbers and the wide confidence intervals of some results. However, interesting contrasts were observed with previous reports and, within the study, between mothers and infants. To our knowledge, this is the first report of the immunological effects of treating helminths in pregnancy and, as such, suggests further important avenues of research.

Our study was designed to examine the effects of active helminth infection through parasitological examination of stool and blood samples, and the effects of albendazole treatment in pregnancy. Examination of a single stool sample is less than 100% sensitive for helminth infection [14], so some infected mothers would be misclassified as uninfected. This would lead to underestimates of helminth effects; thus we expect that the results presented for comparisons between mothers with and without hookworm and their infants are conservative. This misclassification would not affect the validity of the results of the trial (effects of albendazole vs placebo regardless of helminth infection status); or of the sub-group analysis (albendazole vs placebo) in mothers who had positive initial stool results for hookworm.

The most striking effects were observed for maternal hookworm, which was associated with reduced IFN-γ responses to the mycobacterial antigen, CFP, in the mothers, and with increased IFN-γ responses, following BCG immunisation, in their infants. These effects were not explained by measured confounding factors. Confounding factors not measured in this study might include genetic factors, which affect susceptibility to helminth infections [5]. Thus adults found to be helminth-egg-positive might be immuno-genetically different from those found to be egg-negative and immuno-genetic traits transmitted from mother to infant might possibly explain the effects observed. For example, a trait associated with stronger type 1 immune responses might lead to increased susceptibility to hookworm in the mothers and stronger responses to BCG in their infants. However, this explanation seems unlikely: the effects observed were reduced by albendazole treatment in pregnancy suggesting that an effect of active helminth infection may be a more probable explanation of the results.

The suppressive effect observed in mothers was in keeping with the results of studies in laboratory animals indicating suppression of type 1 responses to mycobacteria in the presence of helminths [15-17] and reduced efficacy of BCG against challenge with virulent *Mycobacterium tuberculosis *in mice co-infected with *Schistosoma mansoni *[18]. In humans, studies addressing the effects of helminths on immune responses to mycobacteria (measured as tuberculin skin test responses, or as responses to mycobacterial antigens *in vitro*), have had variable results, perhaps related to choice of comparison groups, or differences in effect by species or intensity of helminth infection [19-22]. In this randomised, placebo-controlled trial, the increase in response among mothers who received albendazole supports a causal role of hookworm in suppression of the response. Studies in both laboratory animals [16,17] and humans [20,23,24], including a placebo-controlled trial of albendazole treatment prior to BCG immunisation among helminth-infected, Ethiopian adults [24], have indicated a suppressive effect of helminths on the immune response elicited by BCG. The contrasting effect of prenatal exposure to maternal hookworm observed in our infants was particularly unexpected in view of the earlier report of a suppressive effect of prenatal exposure schistosomiasis and filariasis on the IFN-γ response following BCG at birth [4]. One possible explanation is that immunological effects differ between helminth species. On the other hand, the earlier study of prenatal exposure did not include uninfected mothers, but compared infants sensitised, or not sensitised to helminth antigens in utero: suppression of IFN-γ responses to mycobacterial antigens following BCG immunisation was observed in the sensitised infants. Thus the nature of the exposure in utero, perhaps related to the species or intensity of helminth infection, may be an important factor.

Our results for IFN-γ could mean that light-to-moderate maternal hookworm infection induced a "better" response to BCG. However, noting that IL-5 responses were more frequent, and BCG scars largest, in the untreated hookworm group, and that scar size correlated with IL-5 production, an alternative hypothesis might be considered. Immunological factors in neonates, such as the elevation of regulatory cytokines suggested by our results for cord blood, may lead to delayed control of BCG replication, a larger "dose" of mycobacteria, and less selective, more destructive cytokine responses, which may, or may not, be protective [25-27]. This hypothesis will be investigated in further studies.

## Conclusion

Maternal helminths, especially hookworm, were associated with suppression of maternal responses to the mycobacterial antigen, CFP, and this effect was largely removed by maternal treatment with albendazole. These results, and the elevated levels of IL-10 in cord blood of mothers with untreated hookworm, were in keeping with current hypotheses regarding the immunoregulatory effects of helminth infections.

By contrast, the association between maternal hookworm and an elevated IFN-γ response to CFP in infants one year after BCG immunisation was unexpected. The mechanism of this effect, and its implications for protective immunity, need to be explored.

## Competing interests

The author(s) declare that they have no competing interests.

## Authors' contributions

AME designed and ran the study, conducted data analyses and wrote the report. PBN and PAM conducted the immunological assays. MN contributed to care of participants. MAQ contributed to study design, statistical analysis, and writing of the report; she conducted the unblinded analysis of the effect of albendazole: all other study staff have remained blinded to treatment status as the study continues. PMN conducted parasitological investigations. MM and JAGW contributed to study design, organisation, interpretation of data and writing of the report.

**Table 1:** Factors associated with high IFN-γ responses to mycobacterial antigens among mothers and their infants.

**Maternal variables in pregnancy**	**Mothers during pregnancy **n = 90		**Infants at one year of age **n = 57	
		
	**Proportion (%) with "high IFN-γ" (> median^a^)**	**P value**	**Proportion (%) with "high IFN-γ" (> median^a^)**	**P value**
Helminths				
none	12/19 (63%)		4/17 (24%)	
hookworm	11/36 (31%)	0.025	16/20 (80%)	0.001
S. mansoni	12/22 (55%)	0.75	7/12 (58%)	0.12
HIV infection				
Negative	39/78 (50%)		26/49 (53%)	
positive	5/12 (42%)	0.76	3/8 (38%)	0.47
Malaria parasitaemia				
Negative	41/77 (53%)		25/49 (51%)	
Positive	3/13 (23%)	0.07	4/8 (50%)	1.00
Age				
< 25 years	28/61 (46%)		19/37 (51%)	
25 or older	16/29 (55%)	0.50	10/20 (50%)	1.00
Education (1 mv)				
none/ primary	29/55 (53%)		19/34 (56%)	
secondary/ tertiary	15/34 (44%)	0.52	10/23 (43%)	0.42
BCG scar *(3 mv)*				
absent	11/28 (39%)		10/14 (71%)	
present	32/59 (54%)	0.25	18/41 (44%)	0.12

^a ^The median level of IFN-γ production in response to CFP for mothers was 69 pg/ml; for infants 206 pg/ml. mv: missing values

## Pre-publication history

The pre-publication history for this paper can be accessed here:


